# Macrophage-Dependent Interleukin-6-Production and Inhibition of *I_K_* Contributes to Acquired QT Prolongation in Lipotoxic Guinea Pig Heart

**DOI:** 10.3390/ijms222011249

**Published:** 2021-10-18

**Authors:** Md. Kamrul Hasan Chowdhury, Laura Martinez-Mateu, Jenny Do, Kelly A. Aromolaran, Javier Saiz, Ademuyiwa S. Aromolaran

**Affiliations:** 1 Nora Eccles Harrison Cardiovascular Research and Training Institute (CVRTI), University of Utah, Salt Lake City, UT 84112, USA; kamrul.chowdhury@utah.edu (M.K.H.C.); Kelly.Aromolaran@utah.edu (K.A.A.); 2 Signal Theory, Communications, Telematic Systems and Computation Department, Telecommunications Engineers School, King Juan Carlos University, 28942 Madrid, Spain; laura.martinez.mateu@urjc.es; 3 Masonic Medical Research Institute, Utica, NY 13501, USA; jennydo1536@gmail.com; 4 Centro de Investigación e Innovación en Bioingeniería, Universitat Politècnica de València, 46022 Valencia, Spain; jsaiz@ci2b.upv.es; 5 Division of Cardiothoracic Surgery & Molecular Medicine Program, University of Utah School of Medicine, Salt Lake City, UT 84112, USA

**Keywords:** guinea pig, ventricular myocytes, lipotoxicity, inflammation, cytokines, interleukin-6, QT prolongation

## Abstract

In the heart, the delayed rectifier K current, *I_K_*, composed of the rapid (*I_Kr_*) and slow (*I_Ks_*) components contributes prominently to normal cardiac repolarization. In lipotoxicity, chronic elevation of pro-inflammatory cytokines may remodel *I_K_*, elevating the risk for ventricular arrythmias and sudden cardiac death. We investigated whether and how the pro-inflammatory interleukin-6 altered *I_K_* in the heart, using electrophysiology to evaluate changes in *I_K_* in adult guinea pig ventricular myocytes. We found that palmitic acid (a potent inducer of lipotoxicity), induced a rapid (~24 h) and significant increase in IL-6 in RAW264.7 cells. PA-diet fed guinea pigs displayed a severely prolonged QT interval when compared to low-fat diet fed controls. Exposure to isoproterenol induced torsade de pointes, and ventricular fibrillation in lipotoxic guinea pigs. Pre-exposure to IL-6 with the soluble IL-6 receptor produced a profound depression of *I_Kr_* and *I_Ks_* densities, prolonged action potential duration, and impaired mitochondrial ATP production. Only with the inhibition of *I_Kr_* did a proarrhythmic phenotype of *I_Ks_* depression emerge, manifested as a further prolongation of action potential duration and QT interval. Our data offer unique mechanistic insights with implications for pathological QT interval in patients and vulnerability to fatal arrhythmias.

## 1. Introduction

Dietary obesity is a major contributor to the increasing prevalence of cardiovascular diseases worldwide [1]. Obesity may have a direct impact on cardiac electrical activity, suggesting that vulnerability to fatal ventricular arrhythmias and sudden cardiac death (SCD) will remain high. The pathology of obesity-related heart diseases [2,3,4,5,6] is associated with cardiac lipotoxicity (or the abnormal accumulation of free fatty acids, FFAs), and enhanced infiltration of activated macrophages. This macrophage activation triggers an increase in the secretion of pro-inflammatory cytokines including interleukin-6 (IL-6), tumor-necrosis factor-alpha (TNF-α), and interleukin-1 (IL-1β) [7,8]. Preventing such lipotoxic effects may inform rationale development of therapeutic interventions for prevention of ventricular arrhythmias in patients. However, the underlying molecular mechanisms are poorly understood. This objective can be advanced by investigating the effects of pro-inflammatory cytokines on ventricular electrical activity and QT interval.

Previous reports have shown that TNF-α decreases the rapid delayed rectifier channels (*I_Kr_*) and the transient outward current (*I_to_*) is inhibited by IL-6 [9] and IL-1β [10], while IL-1β [11] and IL-6 [12], were shown to increase L-type Ca current density. Whether and how the slow component (*I_Ks_*) of the delayed rectifier K current (*I_K_*) is altered by pro-inflammatory cytokines is unknown. We hypothesize that cytokines decrease *I_Ks_*, thereby elevating the risk for fatal arrhythmias and sudden death in metabolic disorders. Our data reveal an additional mechanism for QT interval prolongation.

## 2. Results

### 2.1. Lipotoxicity Induces Pro-Inflammatory Cytokine Release in RAW264.7 Cells

**Figure 1:**
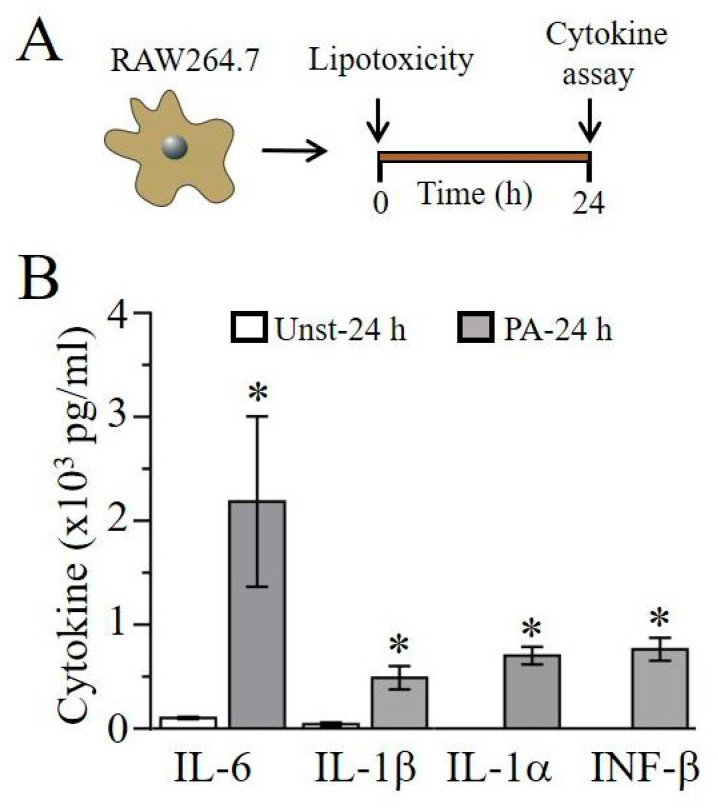
Lipotoxicity induces cytokine secretion in RAW264.7 macrophage cells. (**A**), Schematics of experimental protocol: RAW264.7 macrophage cells were exposed to liptoxicity and different cytokines were assayed and analyzed 24 h after exposure. (**B**), Column graph represents enzyme-linked immunosorbent assay quantification (in pg/mL), of IL-6, IL-1β, IL-1α and INF-β in unstimulated and stimulated cells. Data points are mean ± S.E.M, *n* = 5–10 experiments. * Statistical significance at *P* < 0.05.

First, we assessed the ability of the saturated free fatty acid, palmitic acid (PA, an inducer of lipotoxicity) to induce pro-inflammatory cytokine release in RAW264.7 macrophage cells. We measured cytokine production in the supernatant of untreated and treated (PA-BSA, 1 mM, 24 h Figure 1A) RAW264.7 cells by ELISA kits (R&D systems, Minneapolis, MN). As illustrated in Figure 1B, after being stimulated with PA-BSA for 24 h, we observed a significant production of IL-6, IL-1β, interleukin-1 alpha (IL-1α), and interferon-beta (INF-β) when compared with controls. IL-6 is increased, from a basal level of 101.7 ± 10 pg/mL (*n* = 6) to 2183.96 ± 819 pg/mL (*n* = 6, * *P* < 0.05), with PA-BSA while IL-1β is increased from 42.8 ± 16.8 pg/mL (*n* = 6) to 488.5 ± 112 pg/mL (*n* = 9, * *P* < 0.05). Similarly, the stimulation of RAW264.7 cells with PA-BSA resulted in significant upregulation in IL-1α and INF-1β, both of which showed negligible endogenous amounts. With PA-BSA, IL-1α and INF-1β levels were 701.57 ± 84 pg/mL (*n* = 8) and 762.39 ± 84 pg/mL (*n* = 5), respectively.

### 2.2. IL-6 Induces Ventricular Myocyte Electrical Vulnerability

**Figure 2:**
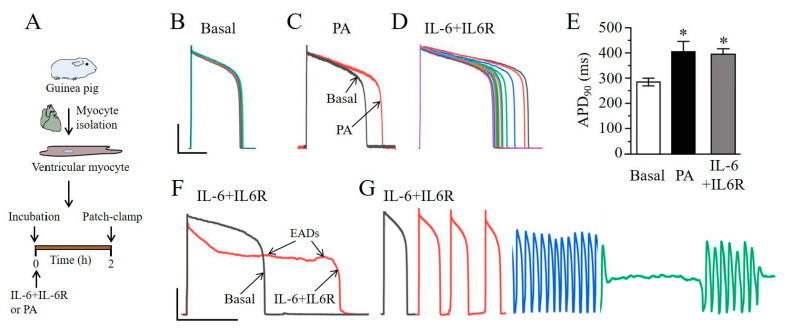
Lipotoxicity and IL-6 + IL-6R promotes arrhythmogenesis in guinea pig ventricular myocytes. (**A**), Cartoon representation of experimental protocol. Guinea pig ventricular myocytes were incubated with PA (1 mM) or IL-6 (20 ng/mL) + IL-6R (25 ng/mL) for 2 h. and ventricular electrical activity was assessed. (**B**), Exemplar superimposed traces (≥ 20 sequential APs) of ventricular action potentials recorded from isolated control or untreated myocytes. (**C**), Representative superimposed AP traces before (Black trace), and after incubation with PA (Red trace). (**D**), Sample APs depicting APD prolongation and beat-to-beat variability in IL-6+IL-6R-treated myocytes compared to untreated myocytes. (**E**), Averaged APD_90_ measured in untreated myocytes, PA- and IL-6+IL-6R-treated cells. IL-6+IL-6R promotes arrhythmogenesis manifested as triggered EADs (**F**), and spontaneous beats (**G**). Data are presented as mean ± SEMs. (Scale bar: 40 mV × 200 ms). * Statistical significance at *P* < 0.05. Data were generated from cardiomyocytes from three different guinea pigs.

We next examined the effect of IL-6 in combination with the IL-6R directly on cardiomyocyte function and determined whether adverse ventricular electrical remodeling occurred in the presence of IL-6 + IL-6R. We assessed the effects of PA (1 mM) or IL-6 (20 ng/mL) + IL-6R (25 ng/mL), at concentrations previously shown to acutely modulate K [13,14], and L-type Ca channels [12,13,15]. Ventricular myocytes were isolated from adult guinea pig hearts and then electrophysiological assays were conducted (Figure 2A). Thus, compared to control (or untreated, Figure 2B), pre-exposure of myocytes to PA (Figure 2C) or IL-6 + IL-6R (Figure 2D) significantly prolonged action potential duration (APD) at 90% repolarization (APD_90_). On average, PA and IL-6 + IL-6R significantly increased APD_90_ by 42% (or from 284.4 ± 15.2 ms, *n* = 26, to 404 ± 41 ms; *n* = 6, * P < 0.05, Figure 2E) and 39% (or from 284.4 ± 15.2 ms, *n* = 26, to 394.9 ± 21.4 ms (*n* = 6), * *P* <0.05, Figure 2E), respectively. Furthermore, we found that myocytes treated with IL-6 + IL-6R displayed early after depolarizations (EADs, Figure 2F) and triggered beats (Figure 2G). Together, our data demonstrate that IL-6 + IL-6R is pro-arrhythmic, possibly by preventing the ability of *I_Kr_* and/or *I_Ks_* to limit repolarization during an action potential.

### 2.3. Q-T Interval Is Prolonged in Lipotoxic Hearts

**Figure 3:**
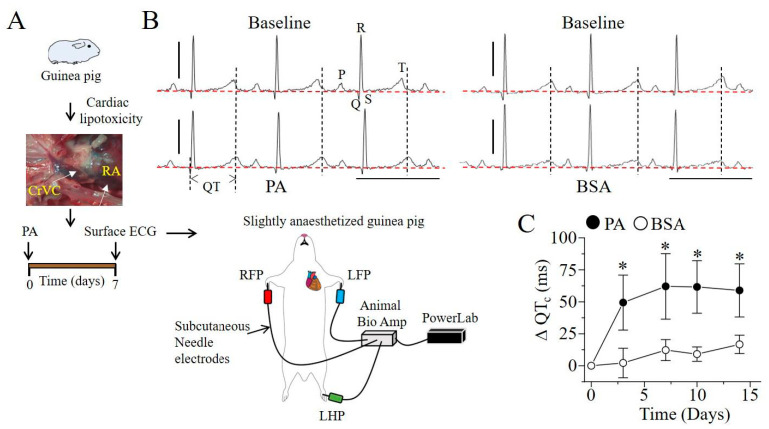
Lipotoxicity induces QT_c_ prolongation in guinea pig. (**A**), Experimental protocol used to assess the effect of lipotoxicity induced by BSA conjugated palmitic acid, injected in guinea pig cranial vena cava (CrVC). QT_c_ was measured at day 0 and progressively after 3, 7, 10 and 14 days. (**B**), Representative traces of surface ECG recorded at baseline and in lipotoxic (PA-BSA), and non-lipotoxic (BSA-alone) guinea pigs at day 7. (**C**), Plot showing progressive changes in QT_c_ relative to time computed from lipotoxic (●) and non-lipotoxic (◯), guinea pigs. Red horizontal lines indicate zero baseline. Compared to non-lipotoxic controls, lipotoxic guinea pigs displayed significant QT_c_ prolongation which peaked after seven days and remained sustained thereafter up until day 14. Data are presented as mean ± SEMs. (Scale bars: 0.5 mV × 200 ms). * Statistical significance at *P* < 0.05.

If lipotoxicity is a potent inducer of IL-6 secretion (Figure 1), and APD is the sole determinant of QT interval in guinea pig, we expect to see rapid and progressive changes in QT interval in lipotoxic guinea pigs. To investigate this, adult guinea pigs were exposed to either 1 mM PA-BSA or BSA alone (controls) through the cranial vena cava (CrVc), and right atrium (RA, Figure 3A). Progressive changes in QT interval were determined in ECG measurements conducted over a period of 14 days (Figure 3A). Figure 3B shows typical ECG traces measured in non-lipotoxic and lipotoxic guinea pigs at day 7.

Interestingly, Figure 3C, confirms our prediction. Thus, compared to non-lipotoxic controls, the change in the heart-rate corrected QT interval (ΔQT_c_) was significantly greater in lipotoxic guinea pigs (Figure 3C). Additionally, ΔQT_c_ rapidly reached a peak at day 7 and remained sustained for an additional seven days. Thus, on average ΔQT_c_ was 12.2 ± 8.20 ms (or 271 ± 8.23 ms vs. 287.5 ± 3.7 ms, * P < 0.05, *n* = 7, open circles, Figure 3C), and 62.1 ± 25.6 ms (or 244 ± 22 ms vs. 303.5 ± 3.7 ms, * P < 0.05, *n* = 8, filled circles, Figure 3C). There were no measurable differences in the heart rate, PR and QRS intervals at baseline or seven-days after exposure to PA-BSA or BSA (Table 1).

Collectively the data suggest that IL-6 may contribute to the initiation of QT prolongation in lipotoxic hearts, while IL-1β, IL-1α and INF-β may act to sustain the QT prolonging effect of IL-6.

**Figure 4:**
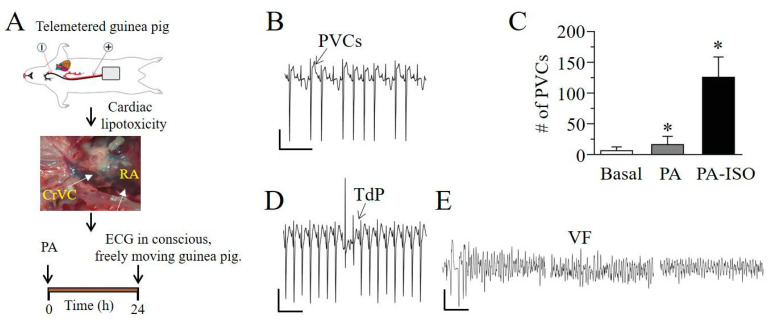
Lipotoxic adult guinea pigs show vulnerability to ventricular arrhythmogenesis. (**A**), Cartoon depiction of experimental protocol. Telemetered guinea pigs were exposed to lipotoxicity and altered cardiac rhythms were monitored for 24 h (**B**), Representative images of cardiac rhythms from adult guinea pigs exposed to ISO demonstrating premature ventricular contractions (PVCs). (**C**), Quantification of PVCs measured in non-lipotoxic, lipotoxic and lipotoxic +ISO adult guinea pigs. Lipotoxic guinea pigs in the absence and presence of ISO displayed significantly more PVCs than age-matched non-lipotoxic controls. *TdP* (**D**) and VF (**E**) in a free-moving lipotoxic guinea pig exposed to ISO. Data are presented as mean ± SEMs. Scale bar: 0.5 mV × 100 ms (**B**), 0.5 mV × 10 s (**D**,**E**). * Statistical significance at *P* < 0.05.

To examine the propensity for ventricular arrhythmias in conscious and freely moving lipotoxic guinea pigs, radiotelemetry ECG transmitters (TR54PB, MT10B, KAHA Sciences, Auckland, New Zealand) were implanted, and ECG abnormalities were progressively (24 h, Figure 4A) monitored. Figure 4B illustrates typical premature ventricular contractions (PVCs) recorded in adult guinea pigs. PVC number was significantly higher in lipotoxic guinea pigs compared to controls (Figure 4C). However, adult lipotoxic guinea pigs that were subsequently exposed to isoproterenol (ISO, 0.5 mg/kg) to reproduce sympathetic stimulation, developed more PVCs than controls or lipotoxic guinea pigs that were not exposed to ISO (Figure 4C). Torsade de pointes (TdP) was readily inducible in ISO-treated lipotoxic guinea pigs (Figure 4D); in one of these guinea pigs, we detected an episode of pre-excited atrial fibrillation, which degenerated into ventricular fibrillation and ultimately to SCD (Figure 4E).

**Figure 5:**
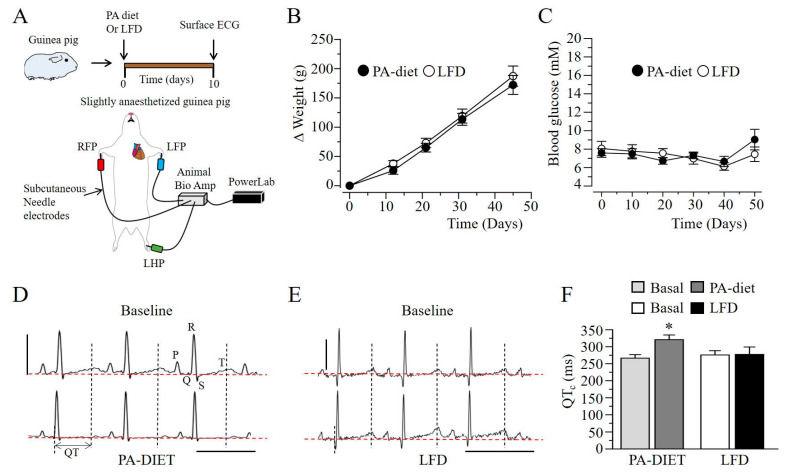
Short-term palmitic acid diet feeding prolongs QT_c_ interval in adult guinea pigs. (**A**), Experimental protocol. Adult guinea pigs were subjected to either PA-diet or low-fat feeding and changes in QT_c_ were assessed at baseline and after 10 days by conducting ECG measurements. Progressive body weight (**B***)* and fasting blood glucose (**C**) changes relative to time measured in guinea pigs fed with either PA-diet (●) or LFD (◯). (**D**), Representative ECG traces measured in a PA-diet fed guinea pig at the beginning and after 10 days, highlighting QT_c_ compared to LFD-fed controls (**E**). Red horizontal lines indicate zero baseline. (**F**), Computed QT_c_ values measured in guinea pigs fed a PA-diet or LFD. Data are presented as mean ± SEMs. Scale bar: 0.5 mV × 400 ms. * Statistical significance at *P* < 0.05.

As a complementary approach, guinea pigs were subjected to short-term (~50 days, Figure 5A), PA-diet and LFD feeding. ECG measurements were conducted to assess relative changes in QT interval. Figure 5B and Figure 5C show that guinea pigs fed either LFD or PA diet displayed similar weights (Figure 5B) and blood glucose levels (Figure 5C), demonstrating a separation of lipotoxicity from hyperglycemia. Adult guinea pigs exposed to PA-diet feeding (Figure 5D) displayed QT_c_ prolongation after 10 days compared to LFD fed controls (Figure 5E), confirming the QT_c_ prolonging effect of lipotoxicity. On average, PA-diet feeding significantly increased basal QT_c_ interval by 20% (266.4 ± 10.3 vs. 320.8 ± 13.8, *n* = 12, * P < 0.05, Figure 5F), but remained essentially unchanged in LFD fed controls (275.7 ± 12.6 vs. 277.3 ± 21.9, *n* = 10, *P > 0.05*, Figure 5F).

### 2.4. I_Kr_ and I_Ks_ Are Severely Depressed by IL-6 + IL-6R in Guinea Pig Ventricular Myocytes

**Figure 6:**
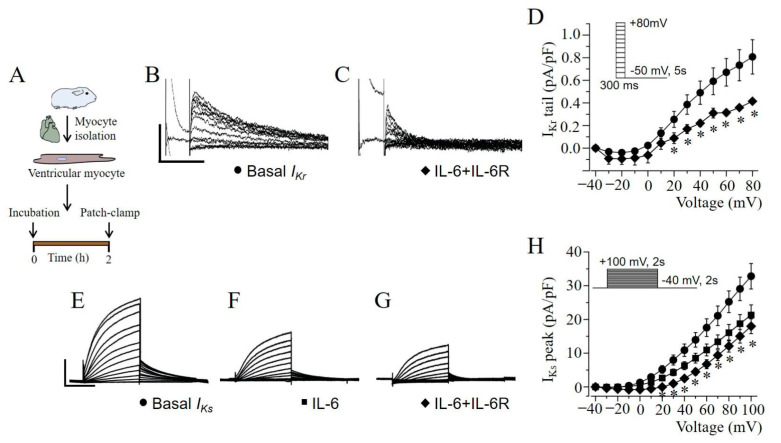
IL-6 inhibits native *I_Kr_* and *I_Ks_* currents in guinea pig ventricular myocytes. (**A**), Experimental protocol: Macroscopic *I_Kr_* and *I_Ks_* currents were measured in freshly isolated ventricular myocytes from adult guinea-pig heart under control (or untreated) conditions and after pre-exposure to IL-6 or IL-6 + IL-6R for 2 h. Exemplar *I_Kr_* tail current traces measured in the absence (**B**), and presence (**C**), of IL-6 (20 ng/mL) + IL-6R (25 ng/mL). (**D**), Population *I-V* curves for *I_Kr_* tail currents. Representative *I_Ks_* current traces measured in the absence (**E**), and presence of IL-6 alone (**F**), or IL-6 + IL-6R (**G**). (**H**), Population current density-voltage curves for *I_Ks_* measured in basal, IL-6- and IL-6 + IL-6R-treated adult guinea-pig ventricular cardiomyocytes. *Insets*: voltage protocols used for evoking *I_Kr_* and *I_Ks_* currents. Data are presented as mean ± SEMs. (Scale bar: 0.5pA/pF × 500 ms and 10pA/pF × 1 s for *I_Kr_* and *I_Ks_* currents respectively). * Statistical significance at *P* < 0.05.

To further probe the mechanisms underlying delayed repolarization (PA and IL-6 + IL-6R, Figure 2), and QT prolongation (PA, Figure 3 and Figure 5), we assessed the effect of IL-6 + IL-6R on cardiac *I_Kr_* and *I_Ks_*, both of which provide outward currents for repolarization [9,16,17]. Electrophysiological experiments were conducted at room temperature (23–25 °C) in adult guinea pig ventricular myocytes (Figure 6A). Figure 6B, shows *I_Kr_* tail currents measured in untreated control myocytes. Pre-exposure (2 h) to IL-6 + IL-6R (Figure 6C) significantly reduced currents at positive potentials to +20 mV. Compared to averaged control values (0.73 ± 0.13 pA/pF, *n* = 13, Figure 6D) measured at +70 mV, *I_Kr_* current density were reduced by 50.7% (or to 0.36 ± 0.01 pA/pF, *n* = 5, * P < 0.05) in the presence of IL-6 + IL-6R.

We next determined whether IL-6 + IL-6R exerted an inhibitory effect on *I_Ks_*. Figure 6E shows typical whole-cell ventricular *I_Ks_* current traces measured in control or untreated adult guinea pig ventricular myocytes. Compared to controls, IL-6 (Figure 6F) or IL-6 + IL-6R (Figure 6G) significantly reduced currents at positive potentials to +10 mV (Figure 6H). Compared to averaged control values (32.8 ± 3.78 pA/pF, *n* = 7, Figure 6H) measured at +100 mV, *I_Ks_* current densities were reduced by 35.1% (or to 21.3 ± 3.00 pA/pF, *n* = 5, Figure 6H, * P < 0.05) in the presence of IL-6 alone, and by 45.2% (17.98 ± 2.19 pA/pF, *n* = 7, * P < 0.05) with IL-6 + IL-6R.

### 2.5. IL-6 + IL-6R Inhibition of Native I_Kr_ and I_Ks_ in Guinea Pig Ventricular Myocytes Is Proarrhythmic in a Human Ventricular Cardiomyocyte In-Silico Model

**Figure 7:**
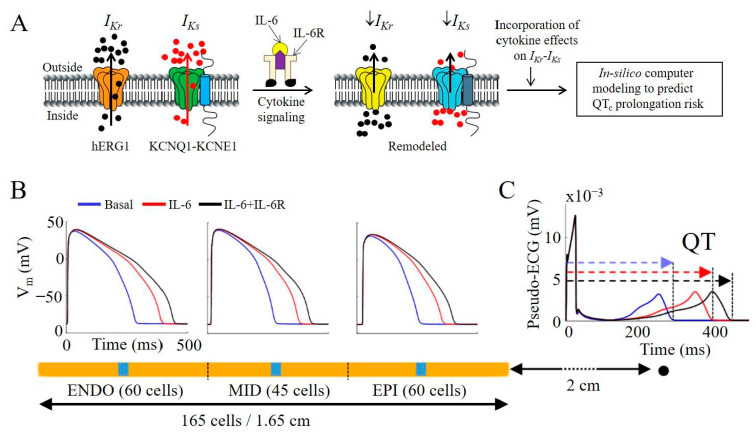
Functional outcome of IL-6-dependent inhibition of *I_Kr_* and *I_Ks_* in simulated electrical activity of human ventricular myocytes. (**A**), Cartoon illustration summarizing the profound inhibition by IL-6 or IL-6 + IL-6R of *I_Kr_* and *I_Ks_* densities, and predicted implications for QT_c_ prolongation and arrhythmogenesis in patients using in silico computer modeling. (**B**), Comparison of AP waveforms in the strand and pseudo-ECG (**C**), calculation in basal conditions, and in the presence of IL-6 and IL-6 + IL-6R. The stimulation begins on the left side of the strand (1st cell in the endocardium) and the pseudo-ECG is calculated at a virtual electrode located at 2 cm from the last cell in the epicardium. The strand is depicted in yellow, while the blue squares over the strand denote cells selected to for AP waveforms.

To determine whether the effects of IL-6 and IL-6 + IL-6R on *I_Kr_* and *I_Ks_* would alter ventricular electrophysiology, we evaluated a human ventricular cardiomyocyte O’Hara model [18], with modifications [19,20] to incorporate our experimental data for IL-6 alone and IL-6 + IL-6R effects on *I_Ks_* (Figure 6H), and previously reported data [9] for *I_Kr_* (Table 2). Computer simulation results are summarized in Table 3 and Figure 7. The depression by IL-6 and IL-6 + IL-6R of *I_Kr_* densities increased APD_90_ by Δ37% and Δ52% (Table 3), respectively, while *I_Ks_* inhibition led to a 3% and 4% increase, respectively (Table 3). Moreover, incorporation of the combined inhibitory IL-6 and IL-6 + IL-6R effects on *I_Kr_* and *I_Ks_* led to a more pronounced effect and increased ΔAPD_90_ to 45% and 65% (Table 4), for IL-6 and IL-6 + IL-6R, respectively, demonstrating an additive effect of IL-6 on *I_Kr_* and *I_Ks_*. The pseudo-ECG measured in strand simulations further revealed the implications on QT interval, and of the regional differences and pathological APD prolonging effect of IL-6 alone or in combination with the IL-6R (Figure 7B). As shown in Figure 7C, QT interval is increased by +36% and +53% (Table 4), for IL-6 and IL-6 + IL-6R, respectively, consistent with the signature high risk proarrhythmic effect that underlie fatal ventricular arrhythmias in patients.

### 2.6. IL-6 Induces Impaired Mitochondrial Metabolism in Human Induced Pluripotent Stem Cell-Derived Cardiomyocytes

**Figure 8:**
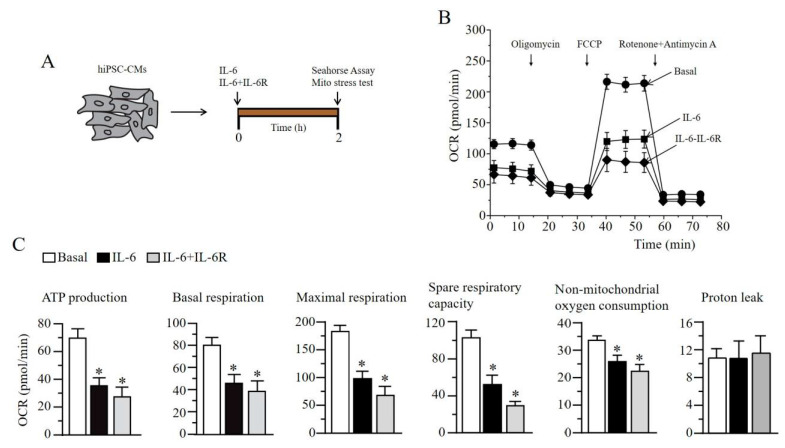
IL-6 and IL-6R leads to impaired mitochondrial bioenergetics in human-induced pluripotent stem cell derived cardiomyocytes. (**A**), Human-induced pluripotent stem-cell-derived cardiomyocytes (hiPSC-CMs) are exposed to IL-6 alone or IL-6 + IL-6R and mitochondrial stress parameters were analyzed after 2 h (**B**), IL-6- or IL-R + IL-6R-treated hiPSC-CMs displayed severely depressed oxygen consumption rate (or OCR), compared to untreated controls, indicating impaired metabolism. (**C**), hiPSC-CMs pre-exposed to IL-6 and IL-6 + IL-6R displayed statistically significant depression in ATP production, basal respiration, and maximal respiration when compared to untreated controls. Nonmitochondrial oxygen consumption respiration and spare respiratory capacity were also severely blunted. Proton leak remained essentially unchanged. Data are presented as mean ± SEMs. * Results with *P* < 0.05 were considered statistically significant.

Finally, we examined the effect of IL-6 on cardiomyocyte metabolism. The heart is critically dependent on OXPHOS, and ion channels are ATP-sensitive and exist in a high density in the sarcolemma [21]. We used the Seahorse XFe56 analyzer Cell Mito Stress Assay to measure the mitochondrial oxygen consumption rate profile in human induced pluripotent stem cell-derived cardiomyocytes (hiPSC-CMs) acutely (2 h), exposed to IL-6 alone or IL-6 + IL-6R (Figure 8A). Pre-exposure to IL-6 alone and IL-6 + IL-6R impaired mitochondrial function (Figure 8B), and severely blunted ATP production, basal and maximal respiration, and spare respiratory capacity (Figure 8C), consistent with the ability of cytokines to modulate mitochondrial metabolic function [22,23,24], suggests a role for pro-inflammatory cytokines in the pathological effects of lipotoxicity on cardiac cell metabolism. Additionally, proton leak was unchanged, suggesting that the mitochondria is not damaged.

## 3. Discussion

Diet related lipotoxicity is a critical contributor to arrhythmias in patients with obesity and related pathologies; however, the underlying molecular mechanisms are unknown. The goal of the present study is to investigate the role for hyperinflammation as a key event in lipotoxicity-induced ventricular electrophysiology remodeling in guinea pig. PA induced significant secretions of distinct pro-inflammatory cytokines (IL-6, IL-1β, IL-1α, and INF-β), in RAW264.7 macrophage cells. PA had a more potent effect on IL-6 production which, in turn, exerted a profound and adverse modulation (APD prolongation, triggered EADs and spontaneous beats, and inhibition of *I_Kr_* and *I_Ks_* densities) of ventricular electrical activity and function. Short-term PA-diet feeding in guinea pig separates hyperlipidemia from significant weight gain and hyperglycemia, induces QT prolongation and promotes cardiac arrythmias (PVCs, TdP and VT). Our data suggests that lipotoxicity-dependent IL-6-production and the subsequent inhibition of *I_Kr_* and *I_Ks_* underlies adverse ventricular electrical remodeling prior to fatal ventricular arrhythmias acquired in obesity and associated disease pathologies.

Macrophages are associated with heightened immune responses to infectious pathogens and tissue damage in obesity [25] and diabetes [26,27]. In metabolic disorders, the enlargement of adipocytes triggers the generation of saturated FFAs through lipolysis [28]. Chronic accumulation of lipid droplets within the myocardium is associated with increased secretion of pro-inflammatory cytokines (IL-6, TNFα, IL-1β) [7,8] and ventricular electrical dysfunction [8].

In cardiomyocytes, repolarizing *I_K_* (*I_Kr_* and *I_Ks_*) function is an important contributor to mechanisms (APD prolongation) [9,29] that predispose to ventricular arrhythmias. Therefore, pathological depression of *I_K_* would be expected to delay repolarization and contribute to ventricular arrythmias. The potential role for impaired ion channel mechanisms in inflammation-related arrhythmias is highlighted by previous studies in patients with inflammatory disorders [30,31,32].

The electrophysiological effects of cytokines on some voltage-gated channels, including *I_Kr_*, have previously been studied by us [9] and others [10,11,33,34,35,36]. However, a paucity of studies in the extant literature has directly assessed the modulation of *I_Ks_* in the heart by pathological cytokine levels. As shown in this study, IL-6 alone or in combination with the IL-6R inhibited *I_K_*_s_ density, and this inhibition increased vulnerability to pathological QT prolongation (Figure 7). Our finding suggests that combined prevention of pathological inhibition of *I_Kr_* and *I_K_*_s_, particularly in lipotoxicity-induced hyperinflammation has the potential to be anti-arrhythmic by shortening APD and reducing fatal ventricular arrhythmias (Figure 7) in patients that show vulnerability to cardiac events. Therefore, it would also be valuable to explore the link between IL-6 + IL-6R, *I_Ks_* and ventricular arrhythmias.

In this study, the β-adrenergic receptor agonist ISO increased the number of PVCs, induced *TdP* and ventricular tachycardia. Furthermore, IL-6 + IL-6R decreased mitochondrial metabolism and, more importantly, ATP production in hiPSC-CMs. Our data suggest that IL-6 may depress *I_Ks_* channel function by limiting available ATP, critical for PKA-dependent phosphorylation of *I_Ks_* channel (KCNQ1) subunits, activation of *I_Ks_* [37,38], and the regulation by β-adrenergic stimulation. This mechanistic insight is likely to have important implications for predicting the effects of pathological changes in cytokines on *I_Ks_* function, and cardiac repolarization especially during exertion.

In conclusion, our data imply that a cytokine-mediated decrease in *I_Ks_* may contribute to high-risk QT prolongation that underlie life-threatening cardiac events. It is intriguing to speculate that dietary interventions that either prevent (1) cardiac and systemic lipid accumulation (anti-lipotoxic drugs promote lipid storage in adipose stores or decrease systemic lipid levels); (2) pathological decreases in *I_Kr_* and *I_Ks_* channel function (cellular mediators that enhance channel opening) may be anti-arrhythmic, and therefore beneficial to obese patients that display vulnerability to arrhythmogenesis and fatal arrhythmias.

## 4. Materials and Methods

### 4.1. RAW264.7 Cell Culture

Low-passage-number RAW264.7 macrophage cells were a kind gift from Dr. Zhiqiang Lin (Masonic Medical Research Institute, Utica, NY, USA). RAW264.7 cells were maintained in DMEM supplemented with 10% FBS and 100 μg/mL penicillin-streptomycin at 37 °C. RAW264.7 cells were pre-exposed to 1 mM BSA alone or palmitic acid-BSA conjugate (20:1 molar ratio) [14] for 24 h. Cytokine production in the supernatant of untreated and treated RAW264.7 cells and human induced pluripotent stem-cell-derived cardiomyocytes (hiPSC-CMs) were assessed by ELISA kits (R&D systems, Minneapolis, MN, USA) according to the manufacturer’s instructions.

### 4.2. Guinea Pig Ventricular Myocytes

Primary ventricular myocytes were isolated and cultured as previously described [39,40]. Briefly, adult male and female Hartley guinea pig hearts were excised and Langendorff perfused with Tyrode solution containing (in mM): 118 NaCl, 4.8 KCl, 1 CaCl_2_, 10 Glucose, 1.25 MgSO_4_, 1.25 K_2_HPO_4_ (pH = 7.4) for 5 min. Ventricular myocytes were isolated by enzymatic digestion in Ca^2+^-free Tyrode solution containing Collagenase B (final concentration, 0.6 mg/mL; Boehringer Mannheim, Indianapolis, IN, USA) for an additional 6 min. The heart was subsequently perfused with high-K solution containing (in mM): 70 KOH, 50 L-glutamic acid (potassium salt), 40 KCl, 10 Taurine, 2 MgCl_2_, 10 Glucose, 10 HEPES, 5 EGTA, and 1% albumin (pH 7.4, with KOH) for 10 min. The digested heart tissue was placed in fresh high-*K* solution, minced into smaller pieces and triturated several times to dissociate the cells. The cell suspension was filtered through a mesh strainer and allowed to settle for 15–20 min. The pellet was resuspended in 10% M199 media and plated on laminin-coated coverslips. Cells were patched 6–8 h after plating.

### 4.3. Preparation of Bovine Serum Albumin Conjugated FFA Solutions

Palmitic acid (PA) stock solution was prepared as previously described [14]. Fatty acid free (20%) bovine serum albumin (BSA, Roche, Indianapolis, IN, USA) was dissolved in Dulbecco Phosphate Buffered Saline (DPBS) and then filtered to sterilize. PA (Sigma-Aldrich, St. Louis, MO, USA) was dissolved in ethanol to generate a 0.2 M fatty acid (FA) stock solution. BSA (20%) and FA (0.2 M) were mixed in 20:1 volume ratio. FA stock solutions (~10 mM) were added directly to M199 culture media or Tyrode’s solution for a final concentration of 1 mM. The vehicle control solution was prepared with BSA, ethanol and DPBS.

### 4.4. Low-Fat Diet and Palmitic-Acid Diet Feeding in Guinea Pig

Guinea pigs (male/female; 200–250 g) were purchased from Charles River Laboratories (Wilmington, MA, USA). Control guinea pigs were fed, ad libitum, a low-fat diet (LFD, Research Diets Inc., New Brunswick, NJ, USA) containing (in kcal%): 10 fat, 70 carbohydrates, 20 protein, and 2300 corn starch. PA diet group was fed diet (in which most of the soybean, was replaced with 315 kcal% palm oil), containing 10% of its kcal from fat, 70% from carbohydrates, and 20% from protein. The PA rich diet contained saturated and unsaturated free fatty acids (FFA), which provided 48.4 and 36.8% of the fat-derived calories. Guinea pigs were fed LFD or palm oil rich diet for a duration of 50 days (~7 weeks), while monitoring temporal changes in weight and blood glucose every 10 days. Blood was taken from the paw and analyzed using an Accucheck glucometer (Roche, Indianapolis, IN, USA).

### 4.5. Preparation of hiPSC-CMs

The hiPSC-CMs (iCell) were purchased from Cellular Dynamics International (CDI cells, Madison, WI, USA). Cells were maintained in media according to the manufacturer’s protocol and instructions. For Seahorse XF Cell Mito Stress Test assay, single hiPSC-CMs were plated onto 96 well Agilent Seahorse XF Cell Culture Microplate, and allowed to recover at 37 °C in a CO_2_ incubator for about 10 days before recordings. Agilent Seahorse XF Cell Mito Stress Test was applied to hiPSC-CMs and oxygen consumption rate (OCR) was measured as a function of time according the manufacturer’s instructions. Cells we exposed to IL-6 (25 ng/mL) alone or in combination with IL-6R (25 ng/mL) for 2 h and six mitochondrial respiration parameters were determined: nonmitochondrial oxygen consumption, respiration, basal respiration, maximal respiration, proton leak, ATP production, and spare respiratory capacity.

### 4.6. Electrophysiology

Whole-cell membrane currents were recorded in cardiomyocytes using an EPC-10 patch clamp amplifier (HEKA Electronics) controlled by PatchMaster software (HEKA) [17,41], or Axopatch-200B amplifier (Axon Instruments, Inc., Burlingame, CA, USA) [9]. A coverslip with adherent myocytes was placed on the glass bottom of a recording chamber (0.7–1 mL in volume) mounted on the stage of an inverted microscope (Eclipse T*i*-U microscope or Diaphot, Nikon, Melville, NY, USA). Micropipettes were from 1.5 mm thin-walled glass and fire-polished. Internal solution contained (in mM): 133 KCl, 0.4 GTP, 10 EGTA, 1 MgSO_4_, 5 K_2_ATP (added on day of experimentation), 0.5 CaCl_2_ and 10 HEPES (pH 7.2). External solution contained (in mM): 147 NaCl, 4 KCl, 2 CaCl_2_ and 10 HEPES (pH 7.4). Pipette resistance was typically 1.5–2 MΩ when filled with internal solution. *I*–*V* curves were generated from a family of step depolarizations (−40 to +100 mV in 10-mV steps for 2 s from a holding potential of −50 mV), followed by a repolarizing step to −40 mV for 2 s to obtain tail currents. Currents were sampled at 20 kHz and filtered at 5 or 10 kHz. Traces were acquired at a repetition interval of 10 s. Cell capacitance or cell size (in pF) was compensated and measured using the built-in compensation unit of the amplifier. Action potential waveforms were continuously recorded from ventricular myocytes in current clamp mode by passing depolarizing currents for 20 ms at a subthreshold (1.5×) intensity in 10 s interval for ~2 min. Ventricular myocytes were pre-treated (2 h) with IL-6 (20 ng/mL) alone or in combination with IL-6R (25 ng/mL) before experimentation.

### 4.7. Electrocardiogram (ECG)

Surface ECG was recorded using a Dual Animal BioAmp amplifier PowerLab (LabChart 8/s, AD instruments, Colorado Springs, CO, USA), and analysis system (LabChart v8.1.2, AD instruments, Colorado Springs, CO, USA). Guinea pigs were placed on a warm pad and subjected to anesthetic inhalation, using a table-top isoflurane (3–4%) vaporizer (Harvard Apparatus, Holliston, MA, USA). After the guinea pig was slightly anesthetized, it was removed, and a mask was used to maintain anesthesia with 1–2% isoflurane (mix of isoflurane and 700 mL O_2_/minute). Anesthesia depth from isoflurane was monitored by respiratory rate and toe pinch response. Electrodes were positioned on the sole of each guinea pig foot. Electrical signals were recorded for 1 min at 1200 Hz, stored on a computer hard disk and analyzed off-line using the average of five representative consecutive beats. Tracings were analyzed for QT_c_ interval, heart rate, PR interval, and QRS duration. QT_c_ interval was calculated by Bazett’s formula where QT_c_ = QT/√RR.

### 4.8. Telemetry

Radiotelemetry ECG guinea pig pressure and biopotential telemeter (SN:11934) transmitters (TR54PB; ZC Analytics, Denver, CO, USA) were implanted under isoflurane anesthesia (2–3% isoflurane in 100% oxygen at 1 L/min). To reduce procedure induced inflammation all animals were given buprenorphine-HCl (0.1 mg/kg, SQ) and carprofen (10 mg/kg/day, SQ) a 1/2 h prior to the time of anesthesia induction. The probes were placed in the abdominal cavity and core body temperature was maintained at 37 °C throughout the procedure, using a heating blanket. The telemetry probes were placed on top of the exposed intestines and the two ECG leads were inserted through the abdominal wall muscles and subcutaneously tunneled in a lead II configuration. The quality of ECG signal and parameters were evaluated during the procedure. Once the quality of the ECG signal was satisfactory the distal ends of the leads were secured to the underlying tissue and the skin incision was closed with 5-0 vicryl and sealed. After the procedure, the guinea pigs were allowed to recover at 37 °C and were monitored until they regained their toe-pinch reflex, ~5–10 min after the end of the anesthesia. Guinea pigs were allowed to recover for 24 h post-surgery before experimentation.

### 4.9. Computer Simulation

We used computer simulations to study the effect of IL-6 alone or in combination with IL-6R in human ventricular myocytes. For this purpose, we used the O’Hara et al. model (ORd) [18], with the modifications proposed by Mora et al. in the fast sodium [19] current and by Dutta et al. in the conductance of several ionic currents [20], to simulate the membrane electrical activity of human ventricular myocytes in basal conditions. The conductance of *I_Kr_* and *I_Ks_* were modified based on the changes measured in experiments with guinea pig ventricular myocytes pre-treated with IL-6 alone or a combination of IL-6+IL-6R. Single cell and heterogeneous 1D strand simulations, as well as pseudo-ECG recordings, were performed by using the ELVIRA software [42], with a time step of 0.01 ms. Unicellular models were stabilized for 60 min and an additional stabilization of 100 pulses was carried out in strand simulations, at a basic cycle length of 1000 ms. The heterogeneous 1D strand was comprised of 165 cells distributed in 60 endocardial, 45 midmyocardial and 60 epicardial cells, similar to prior simulation studies [18,43], and the action potential (AP) propagation was solved by using the monodomain formalism. Pseudo-ECGs were computed using the large volume conductor approximation [44], as a result of the propagation of the AP from the endocardium to the epicardium region in the strand, and were calculated at a virtual electrode located 2 cm from the epicardium [18,44]. The QT interval in the pseudo-ECG was defined as the time between QRS onset and the end of the T wave (cross by 0).

### 4.10. Data and Statistical Analyses

The conditions of each individual group of experiments were blinded to experimenter. Electrophysiological data were analyzed off-line using built in functions in Fitmaster (HEKA), Clampfit and Origin software. Current amplitudes (in pA) were divided by cell size (in pF) and expressed as current densities (pA/pF). Data are reported as means ± S.E.M. Statistical differences were determined using one-way ANOVA with Bonferroni post-hoc analysis or two-tailed unpaired *t* test for comparisons between groups and considered significant at *P <* 0.05.

## Figures and Tables

**Table 1:** ECG Parameters measured in guinea pigs at baseline and after 7 days of exposure to BSA alone or PA-BSA.

	QT_c_ (ms)	Heart Rate (BPM)	PR Interval (ms)	QRS (ms)
Baseline	244 ± 22	280 ± 10.5	53.5 ± 1.68	16.6 ± 0.85
PA- BSA	306.7 ± 6.5 *	279 ± 4.89	56.9 ± 1.78	17.6 ± 0.25
Baseline	271 ± 8.23	281.3 ± 17.6	54.3 ± 1.68	16.4 ± 0.86
BSA	283.4 ± 6.21	281.3 ± 6.19	54.7 ± 2.49	17.3 ± 0.50

* *P* < 0.05.

**Table 2:** Factors applied to the conductances of the *I_Kr_* and *I_Ks_* currents.

Currents	Basal	+IL-6	+IL-6-IL-6R
*I_Kr_*	1	0.54	0.42
*I_Ks_*	1	0.65	0.55

**Table 3:** Effects of inhibition by IL-6 and IL-6 + IL-6 of *I_Kr_* and *I_Ks_* on APD.

	**Basal**	**IL-6** ***I_Kr_* Block**	**IL-6** ***I_Ks_* Block**	**IL-6** ***I_Kr_* + *I_Ks_* Block**
*I_Kr_*	1	0.54	1	0.54
*I_Ks_*	1	1	0.65	0.65
APD_90_ (ms)	262	359	270	380
ΔAPD_90_ (%)		37%	3%	45%
	**Basal**	**IL-6-IL-6R** ***I_Kr_* Block**	**IL-6-IL-6R** ***I_Ks_* Block**	**IL-6-IL-6R** ***I_Kr_* + *I_Ks_* Block**
*I_Kr_*	1	0.42	1	0.42
*I_Ks_*	1	1	0.55	0.55
APD_90_ (ms)	262	398	273	431
ΔAPD_90_ (%)		52%	4%	64%

**Table 4:** Pseudo ECG parameters in basal, IL-6 and IL-6 + IL-6R conditions.

Simulations	Parameters	Basal	IL-6	IL-6-IL-6R
Unicellular	APD_90_ (ms)	264	384	437
	APD prolongation (%)		+45%	+65%
Strand	QT (ms)	297	405	454
	QT prolongation (%)		+36%	+53%

## Data Availability

All relevant data are included within the paper itself.
